# Non-Classical Monocytes and Monocyte Chemoattractant Protein-1 (MCP-1) Correlate with Coronary Artery Calcium Progression in Chronically HIV-1 Infected Adults on Stable Antiretroviral Therapy

**DOI:** 10.1371/journal.pone.0149143

**Published:** 2016-02-11

**Authors:** Nath Zungsontiporn, Raquel R. Tello, Guangxiang Zhang, Brooks I. Mitchell, Matthew Budoff, Kalpana J. Kallianpur, Beau K. Nakamoto, Sheila M. Keating, Philip J. Norris, Lishomwa C. Ndhlovu, Scott A. Souza, Cecilia M. Shikuma, Dominic C. Chow

**Affiliations:** 1 Hawaii Center for AIDS, Department of Medicine, University of Hawaii John A. Burns School of Medicine, Honolulu, Hawaii, United States of America; 2 Department of Tropical Medicine, University of Hawaii John A. Burns School of Medicine, Honolulu, Hawaii, United States of America; 3 Straub Hospital, Honolulu, Hawaii, United States of America; 4 Blood Systems Research Institute, San Francisco, California, United States of America; 5 Department of Laboratory Medicine, University of California, San Francisco, California, United States of America; 6 Department of Medicine, University of California, San Francisco, California, United States of America; 7 Los Angeles Biomedical Research Institute at Harbor-UCLA, Los Angeles, California, United States of America; University of Pittsburgh Center for Vaccine Research, UNITED STATES

## Abstract

**Background:**

Persistent inflammation and immune activation has been hypothesized to contribute to increased prevalence of subclinical atherosclerosis and cardiovascular disease (CVD) risk in patients with chronic HIV infection. In this study, we examined the correlation of peripheral monocyte subsets and soluble biomarkers of inflammation to coronary artery calcium (CAC) progression, as measured by cardiac computed tomography scan.

**Methods:**

We conducted a longitudinal analysis utilizing baseline data of 78 participants with HIV infection on stable antiretroviral therapy (ART) in the Hawaii Aging with HIV-Cardiovascular study who had available baseline monocyte subset analysis as well as CAC measurement at baseline and at 2-year follow up. Monocyte phenotypes were assessed from cryopreserved blood by flow cytometry and plasma was assayed for soluble biomarkers using antibody-coated beads in a high sensitivity Milliplex Luminex platform. Change in CAC over 2 years was analyzed as the primary outcome variable.

**Results:**

Of all monocyte subsets and biomarkers tested, higher non-classical monocyte percentage (ρ = 0.259, p = 0.022), interleukin (IL)-6 (ρ = 0.311, p = 0.012), and monocyte chemoattractant protein (MCP)-1 (ρ = 0.524, p = <0.001) were significantly correlated to higher 2-year CAC progression in unadjusted Spearman’s correlation. Non-classical monocyte percentage (ρ = 0.247, p = 0.039), and MCP-1 (ρ = 0.487, p = <0.001), remained significantly correlated to 2-year CAC progression, while IL-6 was not (ρ = 0.209, p = 0.120) after adjustment for age, hypertension, diabetes mellitus, total/HDL cholesterol ratio, smoking history, and BMI.

**Conclusion:**

The percentage of non-classical monocytes and plasma MCP-1 levels were independently associated with CAC progression and may be related to the progression of atherosclerosis and increased CVD risk associated with chronic HIV infection on stable ART.

## Introduction

Patients with human immunodeficiency virus (HIV) infection, even those with well-suppressed HIV infection on antiretroviral therapy (ART), are at increased risk of cardiovascular disease (CVD) events [1,2]. Paralleling clinical observation, imaging studies have demonstrated increased prevalence of subclinical atherosclerosis among HIV-infected patients [3,4]. Inflammation has been increasingly recognized as a key pathologic process in the development and progression of atherosclerosis [5,6]. As antiretroviral-treated HIV infection remains associated with persistent immune activation and inflammation, these processes are hypothesized to promote atherosclerosis and contribute to increased atherosclerotic cardiovascular disease (ASCVD) risk in HIV-infected patients on ART [7,8]. However, the precise immunologic mechanisms that promote atherosclerosis in these patients remains uncertain.

Monocytes are one of the key cellular components of the innate immune system involved in the development and progression of atherosclerotic plaques [6–9]. Monocyte populations are heterogeneous in nature with differences in the expression of cell surface markers and functional characteristics [9,10]. Currently, monocytes are classified into three subsets on the basis of their CD14 and CD16 surface expression: “classical” (CD14^++^CD16^-^), “intermediate” (CD14^++^CD16^+^) and “non-classical” (CD14^low/+^CD16^++^) subsets [11]. This heterogeneity of monocytes has been implicated in the pathogenesis of atherosclerosis [9,12].

In viremic HIV-infected patients, the expansion of both intermediate and non-classical monocytes has been reported [13]. However, only the expansion of non-classical monocyte persisted during 1 year of treatment with ART [13]. A few studies have evaluated the association between monocyte subsets and atherosclerosis in HIV-infected patients. Among these studies, intermediate monocytes [14] and CD16+ monocytes expressing CX3CR1 [15] have been associated with subclinical atherosclerosis. In addition, our group has observed that a fourth monocyte subset, termed the “transitional” monocytes, characterized by low levels of CD14 and negative CD16 expression (CD14^dim^CD16^-^) was associated with carotid artery intima-media thickness (CIMT) [16,17].

Soluble biomarkers are related to several integral processes of atherosclerosis, including endothelial activation, immune cells recruitment, as well as production of other cytokines and acute phase proteins [5,6]. In patients with HIV infection, CRP and IL-6 has been independently associated with CVD events in some [18,19] but not all studies [20]. Similarly, independent association between monocyte chemoattractant protein (MCP)-1 and subclinical atherosclerosis in HIV-infected patients has been reported inconsistently [21–25]. Thus, the relationship between these biomarkers, traditional CVD risk factors, and atherosclerosis remains uncertain in HIV-infected patients.

In this study, we evaluated the association of monocyte subsets and plasma soluble biomarkers with the progression of subclinical atherosclerosis as measured by coronary artery calcium (CAC) in participants with HIV infection on stable ART. CAC has been demonstrated to correlate with coronary atherosclerotic plaque burden [26] and its progression has been associated with coronary heart disease (CHD) events [27,28].

## Materials and Methods

### Study Participants

This is a longitudinal analysis utilizing the baseline data and 2-year follow up data of participants from the Hawaii Aging with HIV-Cardiovascular (HAHC-CVD) study. In this analysis, we included only participants who had available baseline monocyte subsets analysis and coronary artery calcium (CAC) measurement at baseline and at 2-year follow up. HAHC-CVD is a 5-year longitudinal cohort study of the role of oxidative stress and inflammation in HIV cardiovascular risk. The details of the cohort study design and enrollment have been published previously [29]. Briefly, the study enrolled adults, age ≥ 40 years old with documented HIV infection who were on stable ART for ≥ 3 months, from 2009 to 2012. The study was approved by the Committee on Human Subjects at our institution and was performed in accordance with the Declaration of Helsinki, all International Conference on Harmonization Good Clinical Practice guidelines, and applicable local regulatory requirements and laws. Written informed consents were obtained from all participants.

### Clinical assessment

General medical history with special emphasis on CVD and HIV infection was obtained. Clinical parameters including blood pressure (BP), height, weight, body mass index (BMI), and waist to hip ratio were measured. Smoking was defined as a dichotomous variable of ever smoked or never smoked. Blood tests, including CD4+ T-cell count, HIV RNA, fasting (nothing by mouth for 12 hours) total, high-density lipoprotein (HDL), directly measured low-density lipoprotein (LDL) cholesterol, triglycerides, and glucose were performed.

In this study, hypertension was defined as systolic BP ≥ 140 mmHg, or diastolic BP ≥ 90 mmHg on entry visit, self-reported history of hypertension, or use of anti-hypertensive medications. Diabetes mellitus was defined by a fasting blood sugar (FBS) ≥ 126 mg/dL, 2-hour oral glucose tolerance test (OGTT) ≥ 200 mg/dL, or self-reported history of diabetes mellitus. Ten-year coronary heart disease (CHD) risk was calculated by Framingham risk score (FRS) based on a model comprised of age, gender, total cholesterol, HDL cholesterol, systolic blood pressure, treatment of hypertension and any cigarette smoking in the past month using the National Cholesterol Education Program website (http://hp2010.nhlbihin.net/atpiii/calculator.asp) [30]. Participants with diabetes (as a CVD equivalent) or clinical CVD (history of myocardial infarction, angina, coronary disease-related cardiac surgery, or ischemic stroke) were automatically classified as having 10-year CHD risk by FRS of 20%. Clinical CVD events were adjudicated by 2 physician-researchers (C.M.S and D.C.C.). Undetectable plasma HIV RNA was defined as HIV RNA 50 copies/mL or less.

### Coronary Artery Calcium (CAC) measurements

Computed tomography (CT) examinations for CAC were performed locally in Honolulu, Hawaii at Accuimaging, a free standing imaging center, using a dual source CT (DSCT) scanner (Siemens 64-slice Somatom) following previously published methods [31]. Technical quality assurance and centralized imaging analyses were provided by CT reading center at the Los Angeles Biomedical Research Institute (M. Budoff). A radiologist or cardiologist, who were blinded to clinical data, quantified CAC using an interactive scoring system to calculate Agatston score, with any Agatston score >0 defining the presence of CAC [32]. All participants were scanned twice at entry and after 2 years, with total Agatston score used for all analyses.

### Cell Staining and Flow Cytometric Analysis

**Fig 1:**
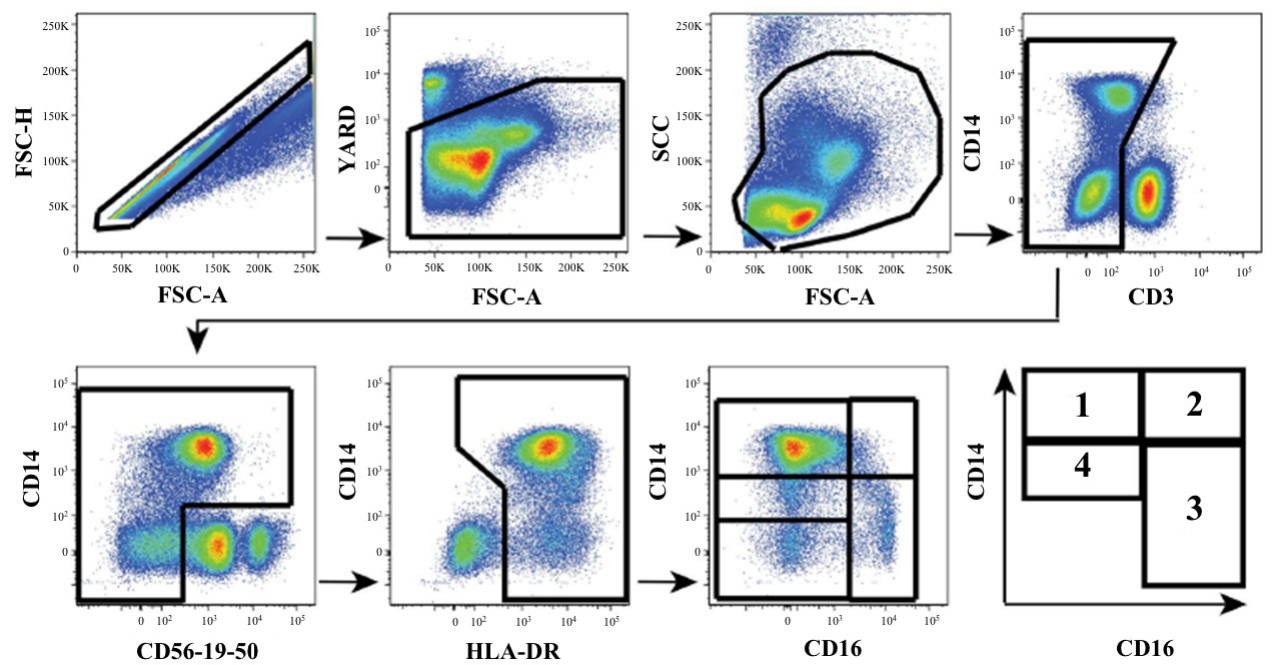
Multiparametric flow cytometry phenotype gating strategy to distinguish distinct monocyte subsets from peripheral blood based on CD16 and CD14 expression. (1) classical monocytes (CD14^++^CD16^-^), (2) intermediate monocytes (CD14^++^CD16^+^), (3) non-classical monocytes (CD14^low/+^CD16^++^), including (4) “transitional” monocytes (CD14^dim^CD16^-^).

In brief, cryopreserved peripheral blood mononuclear cell (PBMC) were thawed and surface-stained with the following antibodies to identify monocyte sub populations as previously described [17]: V500-conjugated anti-CD3, Qdot605-conjugated anti-CD14, Alexa700-conjugated anti-CD16, PE-Cy7-conjugated anti-CD56, PE-Cy7-conjugated anti-CD19, PE-Cy7-conjugated anti-CD20, APC-H7-conjugated HLA-DR monoclonal antibodies (mAbs), All antibodies were from BD Biosciences, except for Q605-conjugated anti-CD14 and yellow Live/Dead (Life Technologies). Stained PBMCs were acquired by flow cytometry, using a 4-laser custom BD-Fortessa instrument (Becton Dickinson) and analyzed with FlowJo software (Treestar Inc Ashland, OR) as previously described [17] (Fig 1).

The total monocyte count was calculated using white blood cell count (WBC) and percent monocyte values on the CBC conducted as part of entry evaluations on the same blood specimen as that utilized for flow cytometry in line with our previous reports [17].

### Soluble biomarkers assessment

Plasma was assayed for soluble biomarkers: soluble E-selectin (sE-selectin), soluble vascular cell adhesion molecule-1 (sVCAM-1), soluble intercellular adhesion molecule-1 (sICAM-1), matrix metalloproteinase (MMP)-9, myeloperoxidase (MPO), C-reactive protein (CRP), serum amyloid A (SAA), serum amyloid P (SAP), IL-1b, IL-6, IL-8, IL-10, tumor necrosis factor (TNF)-α, MCP-1, vascular endothelial growth factor (VEGF), and interferon (IFN)-γ, using antibody-coated beads in a high sensitivity Milliplex Human CVD biomarker panel (Millipore, Billerica, MA) as previously described [23]. The minimum detectable concentration of CRP of this assay is 0.001 ng/mL. Standard curves and samples were tested in duplicate. Samples were acquired on a Labscan 200 analyzer (Luminex, Austin, TX) using Bio-Plex manager software (Bio-Rad, Hercules, CA). The coefficients of variation of all biomarker measurements were less than 10%.

### Statistical analyses

Demographic, cardiovascular, and HIV-related characteristics as well as monocyte subsets, soluble biomarkers, and CAC measurements were described using the median, first quartile (Q1), and third quartile (Q3) for continuous variables and frequency and percent for categorical variables.

Given that 2-year change in CAC is not normally distributed and a substantial percentage of patients had zero 2-year change in CAC, Spearman’s correlation was conducted to evaluate baseline characteristics, including demographic, cardiovascular, and HIV-related characteristics, as well as monocytes subsets and soluble biomarkers for their correlation to 2-year change in CAC.

To further explore the monocyte subsets and soluble biomarkers that were found to be significantly correlated to 2-year change in CAC, we conducted partial Spearman’s correlation with sequential incremental adjustment for 1) Age, 2) Hypertension, 3) Diabetes mellitus, 4) Total/HDL cholesterol ratio, 5) Smoking history, and 6) Body mass index (BMI). Adjusted Spearman’s correlations were conducted using SAS (SAS Institute Inc., Version 9.4, Cary, NC). All other statistical analyses were conducted using SPSS (IBM, Version 21, Armonk, NY). A two-sided probability of p-value less than 0.05 was considered statistically significant.

## Results

### Participant characteristics

Of all participants enrolled into the Hawaii Aging with HIV-Cardiovascular cohort, a total of 78 had available baseline (entry) monocyte subsets analysis as well as coronary artery calcium (CAC) measurement at baseline (entry) and at 2-year follow up and were included in this analysis. Soluble biomarkers at baseline were available in 64 of 78 participants included in this analysis. The demographic, cardiovascular risks, and HIV-related characteristics of the participants are presented in Table 1. Participants were predominantly white (59%) and male (88.5%), with median age of 50.5 years. Almost 8% of patients had a history of clinical CVD events. The median 10-year CHD risk estimated by FRS was 5%. Most participants (84.6%) had undetectable HIV RNA and the median CD4+ T-cell count of all participants was 507.5 cells/mm^3^. Nucleoside Reverse Transcriptase Inhibitors (NRTI), Non-Nucleoside Reverse Transcriptase Inhibitors (NNRTI), Protease Inhibitors (PI), and Integrase Inhibitor (II) were used at entry by 98.7%, 47.4%, 50%, and 11.5% of participants respectively. The baseline monocyte subset percentage and soluble biomarkers are reported as median (Q1, Q3) in Table 1.

**Table 1:** Baseline characteristics, monocyte subset percentage, and soluble biomarkers.

N		78
Age, years [median (Q1, Q3)]		50.50 (46.75, 56.00)
Male gender, n (%)		69 (88.5)
Ethnicity		
	Caucasian, n (%)	46 (59.0)
	African American, n (%)	3 (3.8)
	Native Hawaiian/Pacific Islander, n (%)	10 (12.8)
	Asian, n (%)	4 (5.1)
	Others, n (%)	15 (19.3)
BMI, kg/m^2^ [median (Q1, Q3)]		26.52 (24.47, 28.88)
Hypertension, n (%)		32 (41.0)
Blood pressure		
	Systolic Blood Pressure, mmHg [median (Q1, Q3)]	121.50 (113.75, 130.25)
	Diastolic Blood Pressure, mmHg [median (Q1, Q3)]	75.00 (69.00, 81.25)
Fasting plasma glucose, mg/dL [median (Q1, Q3)]		90.50 (82.00, 95.25)
Diabetes mellitus, n (%)		7 (9.0)
LDL cholesterol, mg/dL [median (Q1, Q3)]		103.00 (82.00, 122.50)
Total/HDL cholesterol ratio [median (Q1, Q3)]		4.00 (3.38, 5.37)
Smoking history, n (%)		48 (61.5)
10-year CHD risk estimated by Framingham risk score, % [median (Q1, Q3)]		5 (3, 15.25)
History of clinical CVD events, n (%)		6 (7.7)
	Myocardial infarction, n (%)	3 (3.8)
	Percutaneous coronary intervention, n (%)	0 (0)
	Coronary artery bypass graft, n (%)	1 (1.3)
	Stroke, n (%)	2 (2.6)
Current Cardiovascular Medications		
	ACEI/ARB, n (%)	14 (17.9)
	Beta blocker, n (%)	8 (10.3)
	Statin, n (%)	17 (21.8)
CD4+ T-cell count, cells/mm^3^ [median (Q1, Q3)]		507.5 (382, 631.25)
CD4+ T-cell nadir, cells/mm^3^ [median (Q1, Q3)] ^a^		150 (50, 245)
Undetectable HIV RNA (≤ 50 copies/mL), n (%)		66 (84.6)
Current Antiretroviral Medications		
	Nucleoside reverse transcriptase inhibitor, n (%)	77 (98.7)
	Non-nucleoside reverse transcriptase inhibitor, n (%)	37 (47.4)
	Protease inhibitor, n (%)	39 (50)
	Integrase Inhibitor, n (%)	9 (11.5)
Hepatitis C infection, n (%)		13 (16.7)
Soluble biomarkers		
	sE-Selectin, ng/mL [median (Q1, Q3)] ^b^	33.88 (22.28, 47.97)
	sVCAM-1, ng/mL [median (Q1, Q3)] ^b^	1122.54 (877.51, 1302.09)
	sICAM-1, ng/mL [median (Q1, Q3)] ^b^	137.54 (112.15, 157.39)
	MMP-9, ng/mL [median (Q1, Q3)] ^b^	51.14 (36.23, 81.34)
	MPO, ng/mL [median (Q1, Q3)] ^b^	16.04 (11.56, 20.96)
	CRP, ng/mL [median (Q1, Q3)] ^b^	8158.20 (3376.22, 30740.73)
	SAA, ng/mL [median (Q1, Q3)]	11649.80 (4397.98, 34788.28)
	SAP, ng/mL [median (Q1, Q3)]	70397.25 (47633.43, 126080.25)
	IL-1b, pg/mL [median (Q1, Q3)] ^b^	0.305 (0.275, 0.310)
	IL-6, pg/mL [median (Q1, Q3)] ^b^	1.67 (1.02, 2.53)
	IL-8, pg/mL [median (Q1, Q3)]	3.56 (2.83, 4.52)
	IL-10, pg/mL [median (Q1, Q3)] ^b^	2.24 (1.20, 4.68)
	TNF-α, pg/mL [median (Q1, Q3)] ^b^	3.17 (1.77, 4.32)
	MCP-1, pg/mL [median (Q1, Q3)] ^b^	137.94 (110.56, 168.00)
	VEGF, pg/mL [median (Q1, Q3)] ^b^	24.15 (13.79, 50.83)
	IFN-γ, pg/mL [median (Q1, Q3)] ^b^	0.78 (0.39, 1.34)
Total monocyte count, cells/L [median (Q1, Q3)]		0.408x10^9^ (0.330 x10^9^, 0.542 x10^9^)
Monocyte subsets		
	Classical monocytes (CD14^++^CD16^-^) percentage [median (Q1, Q3)]	75.33 (70.04, 80.27)
	Intermediate monocytes (CD14^++^CD16^+^) percentage [median (Q1, Q3)]	1.23 (0.52, 3.88)
	Non-classical monocytes (CD14low/+CD16++) percentage [median (Q1, Q3)]	6.15 (4.31, 8.98)
	“Transitional” monocytes (CD14^dim^CD16^-^) percentage [median (Q1, Q3)]	15.05 (10.69, 19.74)

^a^ N = 75

^b^ N = 64

ACEI, angiotensin converting enzyme inhibitor; ARB, angiotensin receptor blocker; CHD, coronary heart disease; CRP, C-reactive protein; CVD, cardiovascular disease; HDL, high-density lipoprotein; IFN-γ, interferon-γ; IL, interleukin; LDL, low-density lipoprotein; MCP-1, monocyte chemoattractant protein-1; MMP-9, matrix metalloproteinase-9; MPO, myeloperoxidase; SAA, serum amyloid A; SAP, serum amyloid P; sE-selectin, soluble E-selectin; sICAM-1, soluble intercellular adhesion molecule-1; sVCAM-1, soluble vascular cell adhesion molecule-1; TNF-α, tumor necrosis factor-α; VEGF, vascular endothelial growth factor.

### Coronary artery calcium (CAC)

The median (Q1, Q3) CAC Agatston score at baseline was 1.87 (0, 44.75) with 50% of participants having zero CAC. At 2-year follow up, the median (Q1, Q3) CAC Agatston score was 3.20 (0, 95.41) with 48.7% of participants having zero CAC. The median (Q1, Q3) 2-year CAC Agatston score progression during 2 years period was 0 (0, 32.715). CAC was increased, unchanged, and decreased in 46.2%, 47.4%, and 6.4% of participants over 2 years.

### Correlation of Participant characteristics, Monocyte subsets, and Soluble biomarkers to 2-year Coronary artery calcium (CAC) progression

In unadjusted Spearman’s correlation (Table 2), older age (ρ = 0.232, p = 0.041), higher body mass index (BMI) (ρ = 0.291, p = 0.010), higher 10-year CHD risk estimated by FRS (ρ = 0.264, p = 0.020), positive history of clinical CVD events (ρ = 0.369, p = 0.001), lower CD4+ T-cell nadir (ρ = -0.254, p = 0.028), and current protease inhibitor (PI) use (ρ = 0.266, p = 0.019), were significantly correlated to higher 2-year CAC Agatston score progression.

**Table 2:** Spearman’s correlation for 2-year coronary artery calcium (CAC) Agatston score progression.

		Correlation Coefficient	p-value
**Age, year**		**0.232**	**0.041**
Male gender		-0.01	0.928
Caucasian ethnicity		0.171	0.133
**Body mass index, kg/m**^**2**^		**0.291**	**0.010**
Hypertension		0.202	0.076
Blood pressure			
	Systolic blood pressure, mmHg	0.163	0.154
	Diastolic blood pressure, mmHg	-0.005	0.966
Fasting plasma glucose, mg/dL		0.055	0.631
Diabetes mellitus		0.113	0.326
Lipid profile			
	LDL Cholesterol, mg/dL	-0.007	0.950
	Total/HDL cholesterol ratio	0.044	0.704
Positive smoking history		0.031	0.791
**Positive history of clinical CVD events**		**0.369**	**0.001**
**10-year CHD risk estimated by Framingham risk score (%)**		**0.264**	**0.020**
Current Cardiovascular Medications			
	ACEI/ARB	0.1	0.381
	Beta blocker	0.207	0.070
	Statin	0.161	0.158
CD4+ T-cell count, cells/mm^3^		-0.185	0.105
**CD4+ T-cell nadir, cells/mm**^**3**^ ^a^		**-0.254**	**0.028**
Undetectable HIV RNA (≤ 50 copies/mL)		0.012	0.919
Current Antiretroviral Medications			
	Nucleoside reverse transcriptase inhibitor	-0.062	0.592
	Non-nucleoside reverse transcriptase inhibitor	-0.121	0.290
	**Protease inhibitor**	**0.266**	**0.019**
	Integrase inhibitor	0.08	0.485
Hepatitis C infection		-0.012	0.916
Soluble biomarkers			
	sE-Selectin, ng/mL ^b^	-0.128	0.315
	sVCAM-1, ng/mL ^b^	0.108	0.397
	sICAM-1, ng/mL ^b^	0.034	0.787
	MMP-9, ng/mL ^b^	0.091	0.476
	MPO, ng/mL ^b^	-0.021	0.869
	CRP, ng/mL ^b^	0.025	0.842
	SAA, ng/mL ^b^	0.089	0.487
	SAP, ng/mL ^b^	-0.021	0.868
	IL-1b, pg/mL ^b^	-0.116	0.362
	**IL-6, pg/mL** ^b^	**0.311**	**0.012**
	IL-8, pg/mL ^b^	-0.090	0.479
	IL-10, pg/mL ^b^	0.001	0.994
	TNF-α, pg/mL ^b^	0.200	0.113
	**MCP-1, pg/mL** ^b^	**0.524**	**<0.001**
	VEGF, pg/mL ^b^	0.090	0.481
	IFN-γ, pg/mL ^b^	0.020	0.878
Monocyte subsets			
	Classical monocytes (CD14^++^CD16^-^) percentage	-0.108	0.348
	Intermediate monocytes (CD14^++^CD16^+^) percentage	0.127	0.269
	**Non-classical monocytes (CD14low/+CD16++) percentage**	**0.259**	**0.022**
	“Transitional” monocytes (CD14^dim^CD16^-^) percentage	-0.037	0.747

^a^ N = 75

^b^ N = 64

ACEI, angiotensin converting enzyme inhibitor; ARB, angiotensin receptor blocker; CHD, coronary heart disease; CRP, C-reactive protein; CVD, cardiovascular disease; HDL, high-density lipoprotein; IFN-γ, interferon-γ; IL, interleukin; LDL, low-density lipoprotein; MCP-1, monocyte chemoattractant protein-1; MMP-9, matrix metalloproteinase-9; MPO, myeloperoxidase; SAA, serum amyloid A; SAP, serum amyloid P; sE-selectin, soluble E-selectin; sICAM-1, soluble intercellular adhesion molecule-1; sVCAM-1, soluble vascular cell adhesion molecule-1; TNF-α, tumor necrosis factor-α; VEGF, vascular endothelial growth factor.

Higher non-classical monocyte percentage (ρ = 0.259, p = 0.022), IL-6 (ρ = 0.311, p = 0.012), and MCP-1 (ρ = 0.524, p = <0.001) were significantly correlated to higher 2-year CAC Agatston score progression in unadjusted Spearman’s correlation (Table 2), while other monocyte subsets and other soluble biomarkers were not.

The correlations of non-classical monocyte percentage, IL-6, and MCP-1 to 2-year CAC Agatston score progression were further evaluated in adjusted Spearman’s correlation. After adjusting for age, hypertension, diabetes mellitus, total/HDL cholesterol ratio, smoking history, and BMI, non-classical monocyte percentage (ρ = 0.247, p = 0.039), and MCP-1 (ρ = 0.487, p = <0.001), remained significantly correlated to 2-year CAC Agatston score progression, while IL-6 was not (ρ = 0.209, p = 0.120) (Table 3). The correlations of non-classical monocyte percentage and MCP-1 to 2-year CAC Agatston score progression were also significant in a model adjusting for 10-year CHD risk estimated by FRS and BMI instead of each individual traditional CVD risk factors (data not shown).

**Table 3:** Adjusted Spearman’s correlation for 2-year coronary artery calcium (CAC) Agatston score progression.

Model	Note	Non-classical monocyte percentage	IL-6 ^a^	MCP-1 ^a^
M0: Univariate	Spearman Rho	0.259	0.311	0.524
	p-value	0.022	0.012	<0.001
M1: M0 + Age	Partial Rho	0.245	0.289	0.525
	p-value	0.032	0.022	<0.001
M2: M1 + Hypertension	Partial Rho	0.239	0.278	0.518
	p-value	0.038	0.029	<0.001
M3: M2 + Diabetes mellitus	Partial Rho	0.239	0.277	0.525
	p-value	0.039	0.031	<0.001
M4: M3 + Total/HDL cholesterol ratio	Partial Rho	0.222	0.258	0.504
	p-value	0.059	0.049	<0.001
M5: M4 + Smoking history	Partial Rho	0.249	0.269	0.499
	p-value	0.037	0.042	<0.001
M6: M5 + Body mass index	Partial Rho	0.247	0.209	0.487
	p-value	0.039	0.120	<0.001

^a^ N = 64

HDL, high-density lipoprotein; IL, interleukin; MCP-1, monocyte chemoattractant protein-1.

## Discussion

Persistent inflammation and immune activation have been hypothesized to promote atherosclerosis in patients with HIV infection [7,8]. In this cohort of HIV-infected participants on stable ART, we found that higher baseline percentage of non-classical monocytes and level of MCP-1, a chemokine that regulates monocytes, were associated with higher 2-year CAC progression independent of traditional CVD risk factors. These associations suggest a potential relation between monocyte-related immune activation and increased CVD risk associated with HIV infection in these patients.

At the site of atherosclerosis, monocytes adhere to the endothelial surface and migrate into the subendothelial space in response to chemokines, such as MCP-1 [8,9,33]. In the subendothelial space, they scavenge oxidized lipid particles, transform into foam cells, and secrete inflammatory cytokines and proteases, such as matrix metalloprotease (MMP), contributing to the progression of atherosclerosis [8,9]. Non-classical monocytes express high level of adhesion molecule expression and exhibit a characteristic “patrolling movement” on the endothelial surface [10,34]. They have been proposed to continually survey the endothelium for signs of damage and inflammation [10,34] and have been demonstrated to extravasate rapidly in response to inflammation in a murine model [35]. It is easy to speculate that these characteristics of non-classical monocytes may facilitate their recruitment into atherosclerotic lesions. In the general population, the expansion of non-classical monocytes has been reported in patients with stable coronary artery disease [36] and non-classical monocyte frequency has been associated with CIMT in renal transplant recipients [37].

The expansion of the non-classical monocyte subset has been reported in HIV-infected patients, even after receiving treatment with ART [13]. Given the aforementioned characteristics of non-classical monocytes and their association with CAC progression observed in our study, it is possible that non-classical monocytes may be actively involved in the pathogenesis of atherosclerosis in HIV-infected patients. However, it remains inconclusive if they contribute to progression of atherosclerosis and increased CVD risk in these patients as there is also some evidence to suggest that they may functionally be atheroprotective, representing a mechanistic defense against increased ASCVD risk [34].

We did not find any associations between other monocyte subsets and CAC progression in this study. Intermediate monocyte subset have been reported to be independently associated with CAC progression in the SUN (Study to Understand the Natural History of HIV/AIDS in the Era of Effective Therapy) study cohort of HIV-infected patients [14]. We have also previously reported an independent association between transitional monocyte subset and CIMT in our entry specimens [16]. The differences observed may be attributable to the limited sample size of our study, difference between subclinical atherosclerosis assessment by CAC and CIMT, and the difference in the characteristics of participants in the two studies.

MCP-1 regulates migration of monocytes via its effect on chemokine receptor CCR2 [33]. It can be produced by several cell types including endothelial cells and monocytes both constitutionally and in response to inflammatory cytokines [33]. Evidence from murine models supports the role of MCP-1 in attracting monocytes to atherosclerotic lesions [38,39]. The association between MCP-1 and CAC progression observed in this study is consistent with a previous report by our group demonstrating a cross-sectional association between MCP-1 and CAC in our entry specimens [23]. Together, the results of both studies suggest that excess CVD risk in HIV-infected patients on ART may be related to increased monocyte recruitment into atherosclerotic plaques. However, the role of MCP-1 in the recruitment of human non-classical monocytes is unclear at present. In a murine study, the lack of CCR2 receptor did not significantly affect the distribution of Ly6C^-^ monocytes (equivalent to human non-classical monocytes) [34].

This study is limited by the small sample size, and the inability of the study to demonstrate cause and effect due to the observational nature of the study. We also did not assess the potential impact on ASCVD risk from other infections such as cytomegalovirus (CMV) co-infection, as well as from translocation of gut microbial products. Host genetics were also not examined in this study. The duration and prior history of specific antiretroviral medications were not extensively analyzed due to the limited number of subjects on each medication. Although PIs as a class of antiretrovirals were associated with increased CAC, no specific PI was significantly correlated with CAC. This was also noted for other antiretroviral classes and specific NRTIs, NNRTIs, and IIs. The lack of a HIV negative comparison group also limited the interpretation of the soluble biomarkers. The ranges to the biomarkers were broad. We compared our values to a set of eighty-six HIV negative controls evaluated in different study populations evaluated by our group and observed that HIV-infected subjects have higher measures of CRP, SAA, and SAP. Median measures were 6.0% higher in SAP, 57.0% higher in CRP, and 69.3% higher in SAA compared to the HIV negative group (data unpublished) [40]. This comparison is consistent with a presentation of increased inflammatory tendencies attributable to HIV infection despite being on stable ART. Despite these limitations, the strength of this study is the careful clinical characterization and CAC measurement performed in the cohort in association with detailed monocyte subsets and biomarker assays. The identification of the non-classical monocyte subset and MCP-1 as being related to CAC progression provides potential new therapeutic targets for intervention in chronic HIV infection.
